# Effects of an antenatal dietary intervention in women with obesity or overweight on child outcomes at 8–10 years of age: LIMIT randomised trial follow-up

**DOI:** 10.1186/s12887-023-04466-4

**Published:** 2023-12-19

**Authors:** Jodie M. Dodd, Andrea R. Deussen, Alexia S. Peña, Megan Mitchell, Jennie Louise

**Affiliations:** 1 Discipline of Obstetrics & Gynaecology, The University of Adelaide, The Robinson Research Institute, Adelaide, South Australia Australia; 2 Women’s and Babies Division, Department of Perinatal Medicine, The Women’s and Children’s Hospital, North Adelaide, 72 King William Road North, Adelaide, South Australia Australia; 3 Discipline of Paediatrics, The University of Adelaide, The Robinson Research Institute, Adelaide, South Australia Australia; 4 Endocrine and Diabetes, The Women’s and Children’s Hospital, North Adelaide, Adelaide, South Australia Australia; 5 Women’s and Children’s Hospital Research Centre, North Adelaide, Adelaide, South Australia Australia; 6 Biostatistics Unit, South Australian Health and Medical Research Institute, Adelaide, South Australia Australia

**Keywords:** Dietary and lifestyle interventions in pregnancy, Follow up of randomised trial, Overweight and obesity, Childhood obesity

## Abstract

**Background:**

The LIMIT randomised controlled trial looked at the effect of a dietary and lifestyle intervention compared with routine antenatal care for pregnant women with overweight and obesity on pregnancy outcomes. While women in the intervention group improved diet and physical activity with a reduction of high birth weight, other outcomes were similar. We have followed the children born to women in this study at birth, 6 and 18 months and 3–5 years of age and now report follow-up of children at 8–10 years of age.

**Methods:**

Children at 8–10 years of age who were born to women who participated in the LIMIT randomised trial, and whose mother provided consent to ongoing follow-up were eligible for inclusion. The primary study endpoint was the incidence of child BMI z-score > 85th centile for child sex and age. Secondary study outcomes included a range of anthropometric measures, neurodevelopment, child dietary intake, and physical activity. Analyses used intention to treat principles according to the treatment group allocated in pregnancy. Outcome assessors were blinded to the allocated treatment group.

**Results:**

We assessed 1,015 (Lifestyle Advice n = 510; Standard Care n = 505) (48%) of the 2,121 eligible children. BMI z-score > 85th percentile was similar for children of women in the dietary Lifestyle Advice Group compared with children of women in the Standard Care Group (Lifestyle Advice 479 (45%) versus Standard Care 507 (48%); adjusted RR (aRR) 0.93; 95% CI 0.82 to 1.06; p = 0.302) as were secondary outcomes. We observed that more than 45% of all the children had a BMI z-score > 85th percentile, consistent with findings from follow-up at earlier time-points, indicating an ongoing risk of overweight and obesity.

**Conclusions:**

Dietary and lifestyle advice for women with overweight and obesity in pregnancy has not reduced the risk of childhood obesity, with children remaining at risk of adolescent and adult obesity. Other strategies are needed to address the risk of overweight and obesity in children including investigation of preconception interventions to assess whether this can modify the effects of maternal pre-pregnancy BMI. The LIMIT randomised controlled trial was registered with the Australian and New Zealand Clinical Trials Registry (ACTRN12607000161426).

**Supplementary Information:**

The online version contains supplementary material available at 10.1186/s12887-023-04466-4.

## Introduction

Childhood obesity is recognised as a significant global health issue, conservatively estimated to affect more than 340 million children and adolescents [1]. In the United States, it is estimated that over 14 million children and adolescents live with obesity, [2] while in Australia, 1 in 4 children or adolescents have a body mass index (BMI) above the 85th centile for age [3].

Approximately 50% of women in Australia commence pregnancy with a BMI above the healthy range [4], and this has been shown to increase the risk of pre-school obesity in her child [5, 6]. There is a body of evidence associating pre-conception obesity and gestational weight gain with increased risks of obesity in children during childhood, adolescence and adulthood [7]. Cohort studies in children have identified early markers of cardiovascular disease including high blood pressure and increased arterial stiffenss in children with BMIs above the 90th centile [8]. There has been focus on clinical interventions during pregnancy to modify a woman’s diet and physical activity to limit gestational weight gain, [9] and provide a potential strategy to break intergenerational obesity risk. This approach has largely targeted women who have overweight or obesity as a high-risk population. The LIMIT randomised trial evaluated such an intervention, in which pregnant women with overweight or obesity were provided with a comprehensive dietary and lifestyle intervention. Women were successful in significantly improving their diet, [10] while reducing the relative risk of high infant birth weight above 4.0 and 4.5 kg by 18% and 41% respectively, although there was no observed effect on maternal gestational weight gain (GWG) [11, 12].

The findings of the LIMIT trial have been confirmed by the UPBEAT trial from the United Kingdom [13], in which 1500 women with a BMI in the obese category were randomised to a similar intervention, finding no difference in weight gain between groups. Systematic reviews [14, 15] and an individual participant data meta-analysis [16] of lifestyle interventions amongst pregnant women of a range of BMIs have found only very small differences in GWG of the order 0.7 to 1.15 kg.

Follow-up of children in the LIMIT Trial has occurred at 6 months, [17] 18 months [18] and 3–5 years of age, [19] showing no evidence of an effect of the intervention. In addition we have reported an independent data meta-anlysis of randomised trials that have evaluated the effects of maternal antenatal dietary and lifestyle intervention on childhood outcomes at 3–5 years of age [20], showing little evidence that pregnancy interventions effect early infant and childhood measures of body composition including adiposity. To our knowledge follow-up assessments have not been reported from older school aged, pre-adolescent children. Our aim was to assess and report the effect of the LIMIT antenatal dietary and lifestyle intervention, on child outcomes at 8–10 years of age and continue our description of this large, high risk cohort. In addition, we have included a measure of arterial stiffness as an early indicator of cardiovascular health [21].

## Methods

The LIMIT randomised controlled trial was registered with the Australian and New Zealand Clinical Trials Registry (ACTRN12607000161426), with the methods and clinical outcomes reported previously [10–12]. Women attending metropolitan public maternity hospitals with a singleton pregnancy and BMI ≥25.0 kg/m^2^ were randomised between 10 and 20 weeks’ gestation to either the Lifestyle Advice Group or to the Standard Care Group [11]. Women who were randomised to the Lifestyle Advice Group were provided with dietary, exercise and behavioural strategies, and goal setting across the course of their pregnancy, [10, 11] consistent with Australian standards, [22, 23] and delivered by a research dietitian and trained research assistants [10, 11]. Women who were randomised to the Standard Care Group received pregnancy care consistent with local hospital guidelines and practices, which did not include advice regarding gestational weight gain.

Following ethics approval and parental written consent, we conducted follow-up of children born to women who participated in the LIMIT randomised trial between 8 and 10 years after birth. Each child assessment was conducted by a trained research assistant who remained blinded to the treatment group allocated at trial entry. Follow-up assessments occurred from May 2019 until May 2021. This time period overlapped with the COVID 19 pandemic, with the ability to attend face-to-face appointments impacted by state-wide lock-downs and restrictions in place at the time.

The primary outcome of this follow-up study was BMI z-score > 85th percentile for child age and sex [24].

A range of secondary outcomes were assessed, including:

Child Anthropometry was assessed by trained research assistants using an established and validated protocol [25, 26]. We measured child’s height, weight, head circumference, bioimpedance measurement, and anthropometric measurements (arm, abdominal and hip circumferences; biceps, triceps, sub-scapular, abdominal, supra-iliac and thigh skinfold thickness). BMI, weight and height were converted to z-scores for age and gender, using WHO standards [24]. Skinfold thickness measures (SFTM) (biceps, triceps, sub-scapular, abdominal and supra-iliac) were also obtained. All anthropometric measurements were done in duplicate and averaged unless greater than 7.5% different in which case the median of three measurements was reported. Measurement of skinfold thickness is considered a reliable and relatively non-invasive method of assessing fat distribution, having been correlated with more invasive measures [27–30]. Fat and lean mass were determined using bioimpedance analysis and calculated using previously published formulae [31, 32].

Blood Pressure: Child blood pressure was measured in a standardised fashion with a blood pressure cuff of appropriate size.

Arterial Stiffness: was assessed via carotid-femoral pulse wave velocity and analysis, using a piezoelectric tonometer (SphygmoCor Device AtCor Medical, NSW) [21].

Child dietary intake and physical activity were assessed via caregiver completed questionnaire. Parents were asked to indicate the number of servings of fruits, vegetables, and dairy consumed daily; the consumption of red meat and processed meats per week; and consumption of non-core foods, including salty snacks, fried potatoes, takeaway foods, soft drinks and other “extra” foods [33].

Physical activity was assessed by caregivers using the Children’s Leisure Activities Study Survey (CLASS) [34]. An activity-specific intensity code from the Compendium of Physical Activities [35] was assigned to each reported activity, and a corresponding estimate of intensity in metabolic equivalent task units (METs) was determined, where 1 MET is equal to the energy expended during quiet sitting [35]. The number of minutes spent in each reported activity was multiplied by its MET intensity, and summed to calculate total daily energy expenditure. Because MET is a measure of intensity and rate of physical activity, the concept of the MET-minute was used to quantify the total amount of physical activity in a comparable way between individuals and across activities [35].

Parents were asked to report the average number of hours their child slept on week days and weekend days and whether their child had a regular bedtime [33].

Child behaviour was assessed using the parent completed Child Behaviour Checklist (CBCL) for 6–18 year olds. The CBCL measures behavioural and emotional functioning and social competence [36]. Responses were summarised into four summary measures: the mean Total Competence Score which sums three scores: activities, social and school; mean of Internalising (internalising behaviours); mean of Externalising (externalising behaviours) and Total Problems (problem behaviours) Scales.

Reading, writing, spelling, grammar and numeracy ability were assessed from the National Assessment Program- Numeracy and Literacy (NAPLAN), [37] a national annual assessment of all students in school years 3, 5, 7 and 9. The test results provide information on how students are performing in the areas of literacy and numeracy and measure their achievements against national minimum standards. Parents provided a copy of their child’s most recent NAPLAN assessment. NAPLAN assessments were not conducted in 2020 due to the COVID 19 pandemic and the associated disruptions to face-to-face student learning.

Pubertal development was assessed by the parent completed Tanner questionnaire [38] which provides pictorial depictions of stages of pubertal development. Stage of pubertal development, and having at least one sign of puberty, were defined using sex-specific criteria according to standards used by the Australian Institute of Family Studies in the Longitudinal Study of Australian children [39].

### Sample size

The available sample size of children at 8 to 10 years of age was predetermined by the LIMIT trial, with a total of 2,212 women randomized [11]. As reported previously, 1,418 children (67% of the eligible cohort) participated in the 3–5 year follow-up assessment [19]. The proportion of children from the Standard Care Group with BMI z-score > 85th percentile at the 3–5 year follow-up was 40% [18]. Assuming a similar rate of participation at the 8–10 year follow-up, this would provide 80% power (with two-sided alpha 0.05) to detect a difference of approximately 6% in the proportion of children with BMI z-score > 85th percentile (proportion in Lifestyle Advice Group from 40 to 34%). For continuous outcomes, there would be 80% power to detect differences of 0.12 standard deviations (SD) between groups.

### Statistical analysis

Data were analysed using intention to treat principles, according to the treatment group to which the woman was randomised in pregnancy. Missing data were imputed using the fully conditional specification (chained equations) method to create 100 complete datasets under the assumption that the data were missing at random (MAR) [40]. All anthropometric and neurodevelopment measures were included in the imputation model, as were all variables used for adjustment (maternal BMI category, study centre, maternal age at trial entry, smoking status, SEIFA IRSD quintile, [41] child sex, and actual age of child at assessment). Additional auxiliary variables from the primary LIMIT trial and from the 6 month, [17] 18 month, [18] and 3–5 year [19] follow-up assessments were also included in the imputation model (Appendix A). No data were available to enable meaningful imputation of missing dietary and physical activity values, NAPLAN scores, pubertal stage, or CBCL so these were analysed using available data only. Data were imputed separately by treatment group. Imputation and analyses of imputed data were undertaken in Stata v15 (StataCorp, Texas), with models fitted to each imputation and estimates combined in the standard manner using Rubin’s rules [40]. The results of imputed analyses were compared with those from complete-case analyses. Further sensitivity analyses were conducted for the primary outcome on the assumption that data were Missing Not At Random (MNAR), assuming both higher and lower incidence of BMI z-scores > 85th percentile as compared with the observed data, in both or only one treatment group.

Continuous outcomes were analysed using linear regression models, with estimates reported as differences in means with 95% confidence interval (CI). Binary outcomes were analysed using log binomial or log Poisson regression models with estimates reported as relative risks (RR) with 95% CI. Both adjusted and unadjusted analyses were performed. Adjusted analyses included stratification variables (maternal early pregnancy BMI category (25.0-29.9 kg/m^2^ vs. ≥ 30.0 kg/m), parity (0 vs. ≥ 1), and centre of planned birth), maternal socioeconomic status (SEIFA IRSD quintile [41]), age, and smoking status. Secondary analyses were performed to test for effect modification by maternal BMI category measured at the time of the woman’s first pregnancy visit. Statistical significance was set at p < 0.05 (two-sided) with no adjustment for multiple comparisons, and all analyses followed a pre-specified statistical analysis plan.

## Results

**Fig. 1:**
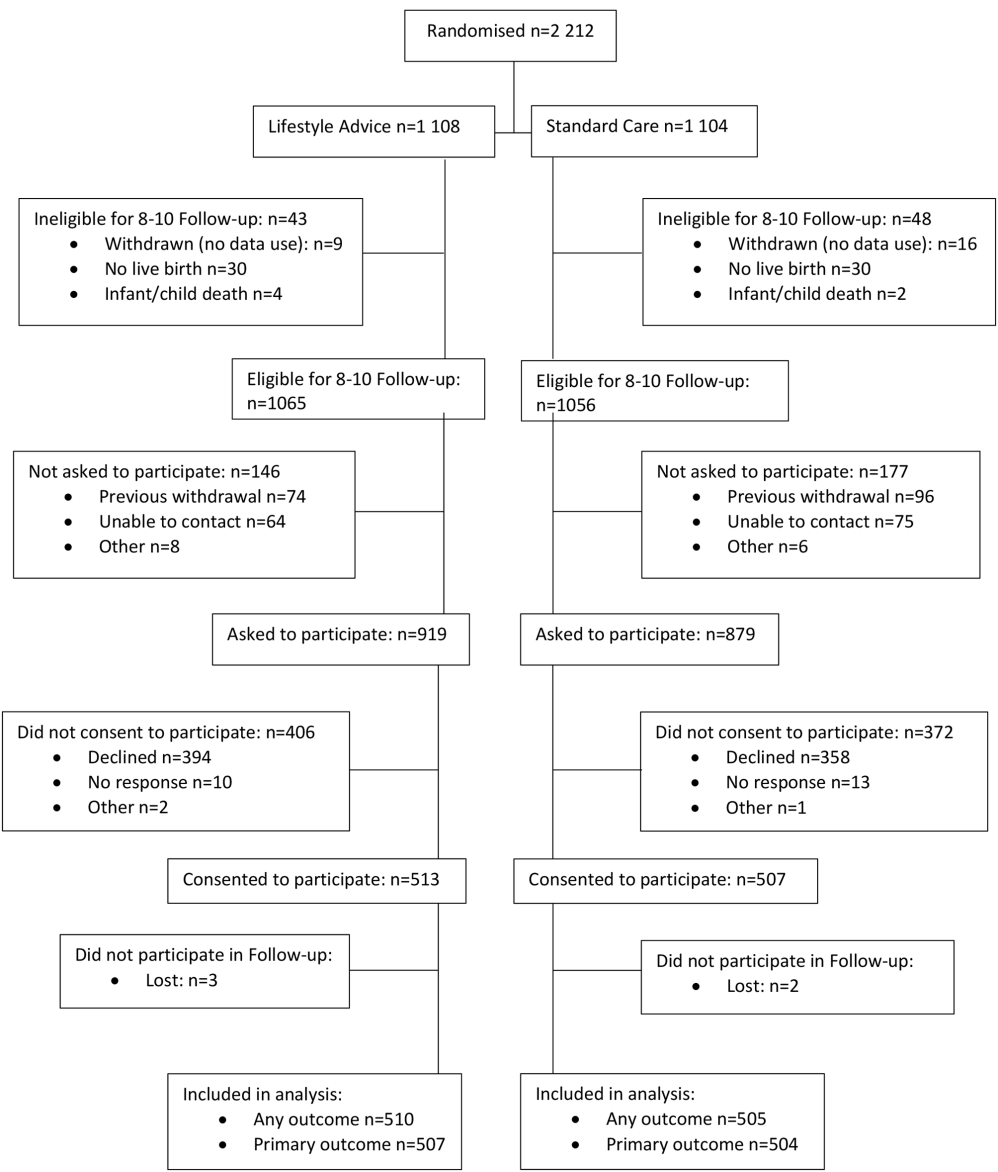
Participant flow

There were 2,121 eligible children of participants in the LIMIT randomised trial contributing data to the imputed analyses (Fig. 1). In total, 1,015 children (Lifestyle Advice n = 510; Standard Care n = 505) (representing 48% of the eligible sample) were assessed at 8 to 10 years of age. Baseline maternal characteristics of eligible child participants (Table 1) were similar to the entire randomised cohort, and similar between treatment groups [11].

**Table 1:** Baseline maternal characteristics from children assessed at 8–10 years of age

Characteristic	Lifestyle Advice N = 510	Standard Care N = 505	Overall N = 1015
Maternal age (years) at trial entry*	30.31 (5.22)	30.09 (5.01)	30.20 (5.11)
Gestational age (weeks) at trial entry^+^	14.21 (12.29, 16.71)	14.29 (12.00, 16.86)	14.29 (12.14, 16.86)
Study Centre^#^			
- Women’s and Children’s Hospital - Flinders Medical Center - Lyell McEwin Health Service	237 (46.47) 161 (31.57) 112 (21.96)	251 (49.70) 141 (27.92) 113 (22.38)	488 (48.08) 302 (29.75) 225 (22.17)
Primiparous at trial entry^#^	219 (42.94)	226 (44.75)	445 (43.84)
BMI (kg/m^2^) at trial entry^+^	30.80 (27.90, 35.75)	30.10 (27.30, 34.70)	30.50 (27.50, 35.20)
BMI (kg/m2) Category#			
- BMI 25.0-29.9 - BMI 30.0-34.9 - BMI 35.0-39.9 - BMI > 40.0	220 (43.14) 149 (29.22) 86 (16.86) 55 (10.78)	245 (48.51) 135 (26.73) 82 (16.24) 43 (8.51)	465 (45.81) 284 (27.98) 168 (16.55) 98 (9.66)
Public patient^#^	497 (97.45)	490 (97.03)	987 (97.24)
Weight (kg) at trial entry*	88.01 (17.32)	86.54 (16.89)	87.28 (17.11)
Height (cm) at trial entry*	164.76 (6.51)	165.15 (6.71)	164.95 (6.61)
Ethnicity^#^			
- Caucasian - Indian - Asian - Other -Missing	470 (92.16) 20 (3.92) 12 (2.35) 4 (0.78) 4 (0.78)	454 (89.90) 17 (3.37) 17 (3.37) 17 (3.37) 0 (0.00)	924 (91.03) 37 (3.65) 29 (2.86) 21 (2.00) 4 (0.39)
Smoking at trial entry^#^	46 (9.02)	42 (8.32)	88 (8.67)
SEIFA IRSD Quintile^# ^^			
- Quintile 1(highest disadvantage) - Quintile 2 - Quintile 3 - Quintile 4 - Quintile 5 (Least disadvantage) - Missing	130 (25.49) 141 (27.65) 90 (17.65) 74 (14.51) 74 (14.51) 1 (0.20)	139 (27.52) 132 (26.14) 72 (14.26) 81 (16.04) 80 (15.84) 1 (0.20)	269 (26.50) 273 (26.90) 162 (15.96) 155 (15.27) 154 (15.17) 2 (0.20)
Child Sex^#^			
- Male - Female	259 (50.78) 251 (49.22)	255 (50.50) 250 (49.50)	514 (50.64) 501 (49.36)
Child Age (months) at Assessment^+^	110.00 (106.18, 117.40)	109.90 (105.97, 117.00)	109.93 (106.06, 117.26)

*= mean and standard deviation

^+^= median and interquartile range

^#^= number and %

^= Socioeconomic index as measured by SEIFA Index of Relative Socio-economic Disadvantage (IRSD) (Australian Bureau of Statistics)

The occurrence of child BMI z-score > 85th percentile was not significantly different between children born to women in the Lifestyle Advice Group, compared with those in the Standard Care Group (p = 0.302, see Table 2), or in children with BMI z-score > 90th percentile (p = 0.446, seeTable 2). There were no statistically significant differences in child weight (p = 0.93, see Table 2), weight z-score (p = 0.98, see Table 2), height (p = 0.64, see Table 2), or height z-score (p = 0.79, see Table 2) (Table 2). There were no statistically significant differences between the treatment groups with regards to body circumference measures or SFTM (Table 2).

**Table 2:** 8–10 year child weight, anthropometric, blood pressure and pulse wave velocity outcomes by treatment group

Outcome	Lifestyle Advice n = 1065	Standard Care n = 1056	Unadjusted Estimate (95% CI)	Unadjusted *p* value	Adjusted Estimate (95% CI)	Adjusted *p* value
BMI z-score > 85th Percentile^#^	479 (45.02)	507 (47.75)	0.94 (0.83, 1.07)	0.379	0.93 (0.82, 1.06)	0.302
Weight (kg)^a^	36.69 (35.87, 37.51)	36.51 (35.74, 37.28)	0.18 (-0.93, 1.30)	0.747	0.05 (-1.02, 1.11)	0.934
Weight z-score	1.02 (0.90, 1.13)	1.00 (0.89, 1.11)	0.02 (-0.14, 0.17)	0.835	0.00 (-0.15, 0.16)	0.984
Height (cm)^a^	137.80 (137.21, 138.39)	137.97 (137.42, 138.52)	-0.17 (-0.97, 0.64)	0.688	-0.17 (-0.90, 0.56)	0.642
Height z-score	0.53 (0.45, 0.60)	0.54 (0.46, 0.62)	-0.01 (-0.12, 0.09)	0.801	-0.01 (-0.12, 0.09)	0.793
BMI (kg/m^2^)^a^	19.04 (18.71, 19.37)	18.88 (18.60, 19.16)	0.16 (-0.28, 0.61)	0.476	0.10 (-0.33, 0.53)	0.651
BMI z-score	0.98 (0.87, 1.09)	0.94 (0.83, 1.05)	0.04 (-0.11, 0.19)	0.603	0.02 (-0.13, 0.17)	0.773
Head Circumference (cm)^a^	53.54 (53.39, 53.69)	53.60 (53.45, 53.74)	-0.06 (-0.26, 0.15)	0.598	-0.05 (-0.25, 0.15)	0.610
Abdomen Circumference (cm)^a^	66.27 (65.40, 67.15)	65.66 (64.75, 66.57)	0.61 (-0.62, 1.85)	0.332	0.45 (-0.75, 1.66)	0.461
Arm Circumference (cm)^a^	22.62 (22.30, 22.94)	22.37 (22.08, 22.66)	0.25 (-0.17, 0.67)	0.245	0.19 (-0.21, 0.60)	0.351
Weight z-score > 85th Percentile^+^	505 (47.40)	528 (50.03)	0.95 (0.83, 1.08)	0.414	0.94 (0.83, 1.07)	0.354
Weight z-score > 90th Percentile^#^	426 (39.96)	437 (41.42)	0.96 (0.84, 1.11)	0.627	0.95 (0.83, 1.10)	0.527
BMI z-score > 90th Percentile^#†^	415 (39.00)	431 (40.8)	0.96 (0.83, 1.11)	0.553	0.95 (0.82, 1.09)	0.446
Biceps SFTM (mm)^a^	10.54 (9.99, 11.09)	10.14 (9.63, 10.65)	0.39 (-0.36, 1.15)	0.306	0.29 (-0.44, 1.03)	0.432
Triceps SFTM (mm)^a^	15.62 (14.91, 16.32)	15.71 (15.12, 16.30)	-0.09 (-0.98, 0.80)	0.837	-0.19 (-1.07, 0.68)	0.666
Subscapular SFTM (mm)^a^	11.25 (10.52, 11.98)	10.89 (10.25, 11.52)	0.36 (-0.59, 1.32)	0.456	0.23 (-0.70, 1.16)	0.623
Supra SFTM (mm)^a^	15.74 (14.66, 16.83)	15.43 (14.41, 16.44)	0.32 (-1.15, 1.79)	0.672	0.15 (-1.30, 1.61)	0.835
Abdomen SFTM (mm)^a^	17.90 (16.80, 18.99)	17.17 (16.13, 18.22)	0.73 (-0.72, 2.17)	0.325	0.55 (-0.88, 1.98)	0.453
Thigh SFTM (mm)^a^	25.63 (24.37, 26.90)	25.32 (24.24, 26.40)	0.31 (-1.33, 1.95)	0.711	0.14 (-1.46, 1.74)	0.867
Sum of SFTM (mm)^a^	95.78 (90.03, 101.52)	91.73 (87.41, 96.06)	4.05 (-3.07, 11.16)	0.265	3.26 (-3.72, 10.24)	0.360
Fat Free Mass (kg)	25.78 (25.24, 26.31)	26.19 (25.69, 26.69)	-0.41 (-1.15, 0.32)	0.270	-0.47 (-1.20, 0.27)	0.215
Total Fat Mass (kg)	10.68 (9.95, 11.40)	10.62 (9.82, 11.42)	0.06 (-0.94, 1.05)	0.907	-0.00 (-0.99, 0.99)	0.996
Percentage Fat Mass (kg)	27.76 (26.49, 29.03)	27.28 (26.11, 28.45)	0.48 (-1.27, 2.22)	0.592	0.41 (-1.33, 2.15)	0.646
Systolic Blood Pressure (mmHg)^a^	108.31 (107.52, 109.10)	108.18 (107.34, 109.02)	0.13 (-0.99, 1.24)	0.823	0.08 (-1.03, 1.19)	0.884
Diastolic Blood Pressure (mmHg)^a^	67.18 (66.44, 67.91)	66.93 (66.13, 67.73)	0.24 (-0.82, 1.31)	0.655	0.26 (-0.81, 1.33)	0.634
Pulse Wave Velocity (m/s)^b^	4.60 (4.46, 4.74)	4.41 (4.30, 4.52)	0.19 (0.01, 0.37)	0.037	0.20 (0.02, 0.37)	0.029

Outcomes are continuous unless otherwise indicated. Descriptives are mean (SD) and estimates are differences in means (Lifestyle Advice – Standard Care) and 95% Confidence Interval from linear regression models

Outcomes marked ‘#’ are binary; descriptives are N (%), with frequencies calculated from estimated proportions combined across imputations. Estimates are Relative Risks and 95% Confidence Interval from log binomial regression models

† Log Poisson regression with robust variance estimation used for adjusted model due to convergence issues with log binomial

Adjusted estimates are from models adjusted for study centre, parity, maternal BMI category, smoking status, maternal age at trial entry and SEIFA Index of Relative Socio-economic Disadvantage (IRSD) quintile

a = adjusted for child sex and actual age at assessment

b = Sensitivity analysis for this outcome was conducted in which three outlier observations (all in Lifestyle Advice Group) were removed; adjusted estimate for this analysis was 0.11 (-0.02, 0.24), p = 0.087

Mean diastolic and mean systolic blood pressure were similar between groups (Table 2).

Pulse wave velocity was significantly higher in the Lifestyle Advice Group compared the Standard Care Group (aMD 0.20; 95% CI 0.02 to 0.37; p = 0.03, see Table 2). A sensitivity analysis in which three outliers were removed, all from the Lifestyle Advice Group, found the adjusted mean difference to be non-significant (aMD 0.11; 95% CI -0.02 to 0.24; p = 0.87, see Table 2).

At 8 to 10 years of age, child energy, fat, protein and carbohydrate intakes were similar across the two treatment groups (Table 3). There were no statistically significant differences with regards to the mean (SD) number of servings per day of fruit (p = 0.81, see Table 3), vegetables (p = 0.75, see Table 3), or dairy (p = 0.37, see Table 3). Similarly, the number of servings per day of “extras” or discretionary foods did not differ significantly between the groups (p = 0.24, see Table 3).

**Table 3:** 8-10year child dietary patterns, physical activity estimates and sleep

Outcome	Lifestyle Advice (n = 510)	Standard Care (n = 505)	Unadjusted Estimate (95% CI)	Unadjusted *p* value	Adjusted Estimate (95% CI)	Adjusted *p* value
Total Energy (kJ)*^a^	7922.01 (2251.58)	7758.01 (2246.91)	163.99 (-115.44, 443.43)	0.250	134.05 (-149.44, 417.53)	0.354
Total Fat (g)* ^a^	67.64 (20.28)	65.77 (19.79)	1.87 (-0.62, 4.36)	0.141	1.61 (-0.91, 4.13)	0.211
Total Saturated Fat (g)* ^a^	28.22 (9.60)	27.25 (9.25)	0.97 (-0.20, 2.14)	0.104	0.87 (-0.32, 2.05)	0.152
Fat as % Total Energy* ^a^	32.23 (3.58)	32.02 (3.87)	0.21 (-0.25, 0.67)	0.372	0.20 (-0.27, 0.67)	0.395
Dietary Fibre (g)* ^a^	28.57 (10.73)	28.55 (11.29)	0.02 (-1.35, 1.39)	0.980	-0.08 (-1.47, 1.31)	0.913
Total Carbohydrates (g)* ^a^	233.80 (72.31)	230.20 (73.27)	3.61 (-5.44, 12.65)	0.434	2.72 (-6.46, 11.91)	0.561
Total Protein (g)* ^a^	74.03 (22.17)	72.04 (21.76)	1.99 (-0.74, 4.72)	0.153	1.68 (-1.08, 4.44)	0.232
Glycaemic Index* ^a^	51.50 (3.44)	51.53 (3.68)	-0.03 (-0.47, 0.41)	0.899	-0.06 (-0.50, 0.38)	0.793
Servings of Vegetables Per Day* ^a^	3.92 (2.36)	3.88 (2.43)	0.05 (-0.25, 0.34)	0.764	0.05 (-0.25, 0.35)	0.746
Servings of Fruit Per Day* ^a^	3.00 (1.96)	3.03 (2.14)	-0.03 (-0.29, 0.22)	0.803	-0.03 (-0.29, 0.23)	0.805
Servings of Dairy per Day* ^a^	2.20 (1.23)	2.12 (1.24)	0.08 (-0.08, 0.23)	0.322	0.07 (-0.08, 0.23)	0.372
Servings of Extras / Discretionary Foods Per Day* ^a^	2.16 (0.86)	2.08 (0.87)	0.08 (-0.03, 0.19)	0.146	0.07 (-0.04, 0.17)	0.237
Total Metabolic Equivalent Tasks (MET Minutes) Per Day* ^a^	8151.20 (3523.18)	8020.63 (3142.57)	130.56 (-284.68, 545.81)	0.537	95.73 (-325.07, 516.54)	0.655
Sleep (hours) on school night Per Day* ^a^	10.15 (0.72)	10.21 (0.72)	-0.06 (-0.15, 0.03)	0.197	-0.06 (-0.15, 0.03)	0.160
Sleep (hours) non school night Per Day* ^a^	10.02 (0.82)	10.03 (0.86)	-0.00 (-0.11, 0.10)	0.934	-0.01 (-0.11, 0.10)	0.891
Regular Bedtime ^# a,b,c^	473/507 (94.41)	471/504 (94.77)	1.00 (0.97, 1.03)	0.803	0.99 (0.96, 1.02)	0.645

* Continuous outcome: descriptives are mean (SD) and estimates are differences in means (Lifestyle Advice - Standard Care) from linear regression model; adjusted models include study centre, parity, maternal BMI category, smoking status, maternal age at trial entry, SEIFA IRSD quintile, infant gender and actual child age at 3–5 year assessment as covariates

# Binary Outcomes: descriptives are N (%) and estimates are Relative Risk of outcome (Lifestyle Advice / Standard Care) from log binomial regression model; adjusted models include study centre, parity, maternal BMI category, smoking status, maternal age at trial entry, SEIFA Index of Relative Socio-economic Disadvantage (IRSD) quintile, infant gender and actual child age at 3–5 year assessment as covariates

a Adjusted for variables listed in (a) plus child sex and actual age at followup

b Adjusted model uses log Poisson regression with robust variance due to nonconvergence of log binomial

c Unadjusted model uses log Poisson regression with robust variance due to nonconvergence of log binomial

Physical activity reported as mean (SD) MET-minutes per week was similar between treatment groups (p = 0.66, see Table 3). The mean duration of sleep overnight on school nights was 10.15 (± 0.72) hours for children in the Lifestyle Advice Group compared with 10.21 h (± 0.72) in the Standard Care Group (p = 0.16, see Table 3). The children slept a similar number of hours on non-school nights with more than 94% in both groups reporting that they have a regular bedtime (Table 3).

Mean scores derived from the CBCL were similar for for Total Competency (p = 0.23, see Table 4); Internalising Scale (p = 0.55, see Table 4); Externalising Scale (p = 0.76, see Table 4) and Total Problems (p = 0.92, see Table 4).

**Table 4:** 8–10 year old Child Behaviour Check List (CBCL)

Outcome Summary measures	Lifestyle Advice (n = 510) Mean (SD)	Standard Care (n = 505) Mean (SD)	Unadjusted Estimate (95% CI)	Unadjusted *p* value	Adjusted Estimate (95% CI)	Adjusted *p* value
Total Competence^a^	22.75 (4.44)	22.39 (4.79)	0.35 (-0.23, 0.94)	0.238	0.36 (-0.22, 0.94)	0.229
Internalising Scale^a^	7.84 (7.17)	7.54 (7.13)	0.30 (-0.59, 1.20)	0.507	0.27 (-0.62, 1.17)	0.545
Externalising Scale^a^	7.59 (7.75)	7.67 (7.61)	-0.08 (-1.04, 0.88)	0.870	-0.15 (-1.10, 0.80)	0.760
Total Problems^a^	28.96 (22.73)	28.90 (23.09)	0.06 (-2.80, 2.92)	0.966	-0.15 (-2.96, 2.66)	0.916

a Adjusted for variables listed in (a) plus child sex and actual age at follow-up

NAPLAN results were available for a smaller proportion of children assessed. Scores for Reading, Writing, Spelling, Grammar and Numeracy were similar between treatment groups (Supplementary Table 1). The number of children with scores for each domain below the minimum national standard were too small for analysis and groups were therefore compared using Fisher’s Exact Test. Scores below minimum national standards for each domain were similar between groups (Supplementary Table 1).

There was no evidence of an effect of dietary and lifestyle intervention in pregnancy on pubertal development of children, as assessed by the parent completed Tanner Questionnaire (Supplementary Table 2).

There was no evidence that the effect of the antenatal intervention was modified by maternal early pregnancy BMI category for any of the reported outcomes (data not shown).

## Discussion

This current study has shown that there were no differences in any anthropometric or behavioural measures of the 8–10 year old children born to women participating in the LIMIT Trial. However the children remain at high risk of childhood obesity with more than 45% of the entire cohort having a BMI above the 85th percentile for their age and sex. Initial findings of the LIMIT trial showed a significant reduction in the proportion of infants with birth weight > 4 kg following the provision of an antenatal intervention. We have followed and reported health outcome on these children at 6 months [17], 18 months [18]and 3–5 years of age [19]. While we have consistently demonstrated an increased risk of obesity amongst this cohort of children, there is no evidence of modification following a dietary and lifestyle intervention during pregnancy.

Our findings are concerning for the future risk of obesity of our cohort of children, and are consistent with other cohort studies reported in the literature. A review of longitudinal cohort studies identified that whilst 70% of adults who have obesity did not have obesity in childhood, approximately 55% of children with obesity will have obesity in adolescence and 80% of adolescents with obesity will have obesity in adulthood [42]. A further study also reports a similar association, whereby 90% of children with obesity will become overweight or obese as adults [43].

There are a number of strengths to our follow-up study. The LIMIT Trial remains the largest RCT to evaluate a diet and lifestyle intervention in pregnant women with overweight and obesity. We have accurately measured early pregnancy weight, height, and BMI; detailed maternal dietary and physical activity history; and consistent provision of the intervention to participants. We have also achieved comprehensive childhood follow-up at multiple time points using robust and well described methodology, including standardised assessment of anthropometric measures, and consistent evaluation of dietary, physical activity, sedentary behaviour, and sleep patterns, all of which are well-recognised early life factors contributing to child overweight and obesity [44].

Our study is not without limitations, including the potential risk of selection bias with data available for 48% of the original LIMIT RCT cohort. However, we do not consider the risk of bias to be significant, with the potential impact on the validity of our findings considered to be low. Baseline and clinical characteristics of women and children for whom data were available and who participated in the follow-up study were similar between the two randomised treatment groups, and similar to the full randomised cohort. (11) We performed analyses through multiple imputation for all children eligible for 8–10 year follow-up (96% of those randomised) to address issues of missing data. The imputation models utilised were robust, incorporating data available from our 6-month [17], 18-month [18] and 3–5 years [19] follow-up assessments. We conducted sensitivity analyses using data imputed under a range of MNAR scenarios, with our results consistent under a variety of plausible assumptions about the magnitude and direction of the difference between missing and observed data.

The dietary assessment did not use a questionnaire that had been validated for this age group, therefore description of the diet quality in this cohort should be viewed with caution. In addition, more accurate assessment of physical activity could have been achieved with comprehensive questionnaires or accelerometers, however costs and participant burden were prohibitive at this scale.

## Conclusions

The findings of this 8–10 year follow up of children from the LIMIT Trial show that the risk of childhood obesity whilst high, was not modified by a dietary and lifestyle intervention in pregnancy. This is further evidence that a continued focus on pregnancy interventions to interrupt the transmission of intergenerational obesity is unlikely to be successful. We would recommend future research effort should be directed at improvements in diet and physical activity prior to conception as a potential target to reduce the risk of overweight and obesity in childhood.

### Electronic supplementary material

Below is the link to the electronic supplementary material.

Supplementary Material 1

## Data Availability

The data reported are available on request as the unrestricted access, including de-identified, is not supported by the local ethics committee. Data access requests can be made to the LIMIT Study Steering Committee via the corresponding author, Professor Jodie Dodd, University of Adelaide, WCH Campus, 72 King William St, North Adelaide South Australia 5006. Approved data access requests will require ethics approval through the Women’s and Children’s Health Network Human Research Ethics Committee, 72 King William St, North Adelaide, South Australia, 5006 (HealthWCHNResearch@sa.gov.au).

## Abbreviations

aRR: Adjusted relative risk
aMD: Adjusted mean difference
BMI: Body mass index
CBCL: Child Behaviour Checklist
CI: Confidence interval
CLASS: Children’s Leisure Activity Study Survey
GWG: Gestational weight gain
MAR: Missing at random
MET: Metabolic equivalent task
NMAR: Missing not at random
NAPLAN: National Assessment Program – Language and Numeracy
SD: Standard deviation
SEIFA IRSD: Socio-economic index of advantage – index of relative disadvantage
SFTM: Skinfold thickness
