# Supplementary material for: Effects of an antenatal dietary intervention in women with obesity or overweight on child outcomes at 8–10 years of age: LIMIT randomised trial follow-up
Source: BMC Pediatr. 2023 Dec 19;23:643. doi: 10.1186/s12887-023-04466-4 (PMC10729523; doi:10.1186/s12887-023-04466-4)
Supplement: Supplementary file 1 — Supplementary Material 1 [file 12887_2023_4466_MOESM1_ESM.docx]

**SUPPLEMENTARY TABLE 1:** 8-10 year old NAPLAN assessment

| **Outcome** | **Lifestyle Advice**  **n= 507** | **Standard Care**  **n = 504** | **Unadjusted Estimate (95% CI)** | **Unadjusted p value** | **Adjusted Estimate (95% CI)** | **Adjusted p value** |
| --- | --- | --- | --- | --- | --- | --- |
| NAPLAN Reading Score*^,a^ | 0.35 (1.19) | 0.17 (1.09) | 0.18 (-0.11, 0.47) | 0.219 | 0.10 (-0.20, 0.40) | 0.513 |
| NAPLAN Writing Score*^,a^ | 0.06 (0.83) | -0.06 (0.78) | 0.11 (-0.09, 0.32) | 0.276 | 0.09 (-0.12, 0.31) | 0.382 |
| NAPLAN Spelling Score*^,a^ | 0.09 (0.90) | 0.13 (0.86) | -0.04 (-0.27, 0.18) | 0.706 | -0.02 (-0.26, 0.22) | 0.864 |
| NAPLAN Grammar Score*^,a^ | 0.25 (0.86) | 0.21 (1.04) | 0.04 (-0.21, 0.28) | 0.774 | 0.02 (-0.23, 0.27) | 0.876 |
| NAPLAN Numeracy Score*,^a^ | 0.19 (0.90) | 0.18 (0.97) | 0.01 (-0.23, 0.25) | 0.942 | 0.05 (-0.20, 0.30) | 0.713 |
| NAPLAN Reading Below Minimum^#,b^ | 1/115 (0.87) | 1/119 (0.84) | – | >0.99 | – |  |
| NAPLAN Writing Below Minimum^#,b^ | 2/114 (1.75) | 1/119 (0.84) | – | 0.615 | – |  |
| NAPLAN Spelling Below Minimum^#,b^ | 3/114 (2.63) | 0/118 (0.00) | – | 0.117 | – |  |
| NAPLAN Grammar Below Minimum^#,b^ | 2/115 (1.74) | 2/119 (1.68) | – | >0.99 | – |  |
| NAPLAN Numeracy Below Minimum^#,b^ | 2/113 (1.77) | 3/120 (2.50) | – | >0.99 | – |  |

^a^ Adjusted for variables listed in (a) plus child sex

^b^ Numbers too small for analysis; Fisher’s Exact test used to compare groups

**SUPPLEMENTARY TABLE 2:** 8-10 year old Stages of Pubertal Development

| **Outcome** | **Lifestyle Advice**  **n (%)** | **Standard Care**  **n (%)** | **Unadjusted Estimate (95% CI)** | **Unadjusted p value** | **Adjusted Estimate (95% CI)** | **Adjusted p value** |
| --- | --- | --- | --- | --- | --- | --- |
| Any Sign of Puberty^a,b^ | 404/507 (80.96) | 406/504 (81.69) | 0.99 (0.93, 1.05) | 0.768 | 0.99 (0.93, 1.05) | 0.747 |
| Stage of Pubertal Development ^a,c^ |  |  |  |  |  |  |
| -Pre-pubertal | 276/499 (55.31) | 282/497 (56.74) | 1.07 (0.84, 1.36) | 0.577 | 1.08 (0.85, 1.38) | 0.531 |
| -Early Pubertal | 132/499 (26.45) | 129/497 (25.96) |  |  |  |  |
| -Mid-Pubertal | 84/499 (16.83) | 86/497 (17.30) |  |  |  |  |
| -Late Pubertal | 7/499 (1.40) | 0/497 (0.00) |  |  |  |  |

a Adjusted for variables listed in (a) plus actual age at followup

b Adjusted model uses log Poisson regression with robust variance due to non-convergence of log binomial

c Analysed using ordinal logistic regression; estimate is OR of being in lower categories vs higher categories
